# Psychological first aid in a cross-border medical emergency drill: Detected barriers, perceptions, and opportunities for improvement

**DOI:** 10.3934/publichealth.2025049

**Published:** 2025-10-10

**Authors:** Olga Malas, Andrés Cuartero, Laia Reales, Judit Cativiela, Yuri Lázaro

**Affiliations:** Medical Emergency System, Departament de Sanitat i Seguretat Social, Departament de Salut, Generalitat de Catalunya, Travessera de les Corts, 131–159, 08028 Barcelona, Spain

**Keywords:** psychological first aid, cross-border disaster, drill, simulation-based training, mass casualty incident

## Abstract

Disasters, especially cross-border ones, represent a significant challenge for public health, with potential repercussions on the mental health of both the victims and the first responders. In this context, Psychological First Aid (PFA) interventions in a safe area near the disaster site are essential to mitigate an acute emotional impact and prevent the onset of subsequent psychological disorders. In this context, the aim of this study is to evaluate the implementation and management of a PFA protocol in an international collaboration setting, thus exploring not only the effectiveness of the training but also the coordination of these protocols in emergency situations within a multi-agent, multicultural environment. A cross-border mass casualty incident simulation was conducted, which involved emergency medical services from Spain, France, and Andorra. Agreed cross-border disaster response protocols were implemented, including the PFA protocol, which was implemented by a Psychological Emergency Unit (PEU). Both qualitative and quantitative data collection and analysis techniques were employed. Strengths, such as the clinical and ethical response, were observed for the PEU performance, though operational and coordination shortcomings were also identified, including a critical weakness in the planning phase where the need for PFA for healthcare personnel was not anticipated, thus preventing its implementation for this group. Cross-border coordination faced challenges due to language barriers, thus underscoring the importance of interpreter availability and intercultural mediation in multinational emergency responses. Strengths and weaknesses were analyzed, and suggestions for improvement were proposed. The simulation provided valuable insights into the performance and applicability of tested PFA protocols in a cross-border context. Strengthening training and operational planning will be key to optimizing the response in real-life situations.

## 1. Introduction

Disaster management is of vital importance in the field of Public Health due to its profound human, social, and economic repercussions [1]. A disaster is defined as a sudden event that causes material damage and physical injuries that exceed the response capacity of local resources, including the demand for psychological support for survivors and frontline personnel [2]. This is because disasters are critical, sudden, and powerful incidents that fall outside the range of ordinary human experiences and generate intense emotional responses in those who experience them [3]. As a result, both victims and frontline personnel often exhibit high levels of stress due to direct exposure to the event [4], which has been linked to disorders such as post-traumatic stress disorder (PTSD) and depression [5],[6], even many years after the traumatic event [7]. A recent meta-analysis reported that the average rate of PTSD among paramedics was 11% and the rate of depression was 15%, as compared to a prevalence of PTSD in the general population of 2.3% and depressive symptoms of 8.1% [5].

Given their proven effectiveness in preventing subsequent psychopathological issues [8], the implementation of Psychological First Aid (PFA) protocols is recommended, with a particular emphasis on emergency personnel [8],[9].

### 1.1. Psychological first aid (PFA)

There is a broad consensus on the value of PFA as an early intervention following a disaster, both for the victims and for the frontline response personnel [10],[11]. A key characteristic of PFA is that it can be administered by both mental health professionals and other disaster response personnel [12], as they do not require a clinical diagnosis or psychological treatment, which are reserved for qualified psychologists [13].

Although there are several models of PFA, the World Health Organization (WHO) model is widely recognized for its three core principles: ‘Look, Listen and Link’ [14]. These principles guide responders to observe an individuals' needs, listen to their concerns, and connect them with appropriate support. Additionally, Hobfoll et al. [15] identified five essential elements for immediate and mid-term mass trauma intervention—promoting safety, calming, self- and community efficacy, connectedness, and hope—which provide an empirical foundation that informs many PFA frameworks. Additionally, it is important to note that other organizations, such as the International Federation of Red Cross (IFRC) and Red Crescent Societies, have developed their own PFA guides, thus reflecting variations in approach adapted to different contexts [16].

In practice, the most common approach is to focus on listening, facilitate social support, take direct actions [17],[18] to reduce high stress levels [19], comfort the victim [17], and mobilize the necessary resources to assist those affected [17]–[19].

For more detailed information on PFA, readers may consult the WHO's PFA Guide [14], the Johns Hopkins PFA Guide [20], and the Guide for Attention to People in the Face of Serious Emergencies in Catalonia (APCAT) [21], which was the framework tested in this study.

### 1.2. Cross-border disasters

The health risk associated with a disaster is directly linked to the effectiveness of planning, and the effectiveness of such planning directly depends on the assumptions on which it is based [22]. These assumptions include the following: the immediate notification and timely arrival of emergency personnel at the disaster site; an effective search for affected individuals; triage and initial care on site; the appropriate distribution of casualties among care centers; and clear communication between all parties involved and healthcare facilities [22]. Therefore, the preparation for disaster interventions must be holistic, thereby encompassing the joint development of response protocols, coordinated planning, comprehensive preparation, and inter-organizational training, so that emergency services, healthcare providers, and relevant agencies can operate as a single, unified response unit [8].

This becomes particularly complex when a disaster affects more than one country, as is the case of cross-border disasters. Effective planning for intervention in such scenarios requires operational agreements and the overcoming of legal and communication barriers [23]. In Europe, although cultural and linguistic differences have a statistically significant negative effect, administrative and legislative obstacles most severely hinder the efficient response to and management of cross-border disasters [24].

Furthermore, disaster education and training across countries shows considerable variability and generally lacks a competency-based approach [23],[25]. While countries such as Spain have Psychological Emergency Units (PEUs) integrated into their emergency medical systems, which are responsible for providing psychological first aid at safe area nearby to the disaster site, other neighboring countries lack such an integration.

To address these types of issues, recent years have seen the development of initiatives such as the Egalurg Project (https://egalurg.es/), which aims to reduce bureaucratic obstacles and establish jointly agreed intervention protocols between neighboring countries.

### 1.3. Simulation-based training techniques

Numerous studies underscore the educational value of simulations, especially in developing both technical and non-technical healthcare skills [26]. Simulations have proven particularly effective in improving leadership, communication, and coordination, which are areas often identified as weaknesses among emergency and disaster personnel [27]. Furthermore, they provide a safe, controlled environment in which to replicate high-risk disaster scenarios without endangering either simulated casualties or learners [28].

Emergency medical and rescue services are typically required to provide regular training to their personnel, and simulation exercises are commonly used to enhance coordination and interoperability between teams [27]. However, the participation of PEU's in such simulations is a relatively recent development. Challenges in training PEU staff, particularly their limited understanding of their roles within multidisciplinary disaster response teams, have been identified as a gap in the literature [29].

This gap motivated the development of the simulation-based training program described in this study.

### 1.4. Contextualisation of this study

France and Spain share a 656 km border defined by the Pyrenees range, and the Principality of Andorra is nestled between them. This geographical situation poses challenges for land-based communication between the countries and may limit access to healthcare for the population in the event of emergencies or disasters. Within the framework of the EGALURG Project [from the French words *égalité* (equality) and *urgence* (emergency)], a cross-border cooperation network has been established between France, Spain, and Andorra to improve healthcare provision in this region. The network seeks to overcome legal barriers to cross-border healthcare collaboration and to provide shared specialized trainings. Moreover, an information technology (IT) platform was developed (referred to as GA-IMV -from Spanish: *Gestión de Afectados en Incidente de Múltiples Víctimas* – Management of Affected Persons in Multiple-Victim Incidents) to register victim data during triage (including real-time Spanish/French translation) and to support patient referral to appropriate healthcare centers during mass casualty incidents.

The simulation exercise described in this study took place in Spain, on the border with France. Consequently, the incident management was led by Spain's Medical Emergency Services (MES). Since 1985, Spanish law has recognized the right of citizens, including first responders, victims, and their families, to receive psychological care in disaster situations [30]. In response, protocols have been implemented to ensure that comprehensive health support is provided to both direct and indirect victims of disasters. The APCAT protocol (from Spanish: *Atención a las Personas en Cataluña* – Care for People in Catalonia, for use in situations of serious emergencies). was applied where this simulation was performed [21].

According APCAT protocols [21], the MES is responsible for coordinating and delivering this care. It operates with Medical Emergency Units (MEUs) and PEUs, which are tasked with assisting victims in a safe area nearby or in close proximity to the disaster site or incident. Psychological support in emergencies is usually provided in cold zones, which are away from the main area of operations, because warm and hot zones are too dangerous. However, the APCAT protocol allows PEUs to work in warm zones together with MEUs, as long as the area has been secured and declared safe by the incident commander.

As part of the APCAT protocols [21], the PEU protocol clearly defines the roles of psychologists according to the intervention area (nearby area and other areas). At the safe area nearby of the disaster site, they fulfil a dual role: monitoring and supporting the mental health of rescue personnel, and conducting psychological triage and the registration of victims. It does not include psychological assistance to victims within the disaster area, which will be provided outside of it, at either Care Areas for Affected Persons or at health centers.

Psychologists, alongside other medical professionals, apply the Simple Triage and Rapid Treatment (START) system to perform triage. The victims were triaged using the internationally recognized color code system (black, red, yellow, green), which indicates the severity of injuries and the urgency of the required medical care. After triage, the victims were physically relocated to designated care areas identified by the same color codes. These zones (“black zone”, “red zone”, “yellow zone”, and “green zone”) were established at the incident site to group individuals according to their clinical priority. This spatial organization of victims enabled better coordination of medical and psychological support teams, thereby facilitating targeted interventions according to the needs of each group. Therefore, although the triage colors usually refer to the clinical status rather than the physical location, in this operational context, they were also used to identify the layout of the response system.

As individuals suffering from psychological trauma without physical injuries are assigned to the GREEN zone, psychologists predominantly operate within this area, though not exclusively. According to the protocol, only those exhibiting severe mental health symptoms or significant behavioral disturbances are eligible for on-site psychological care. In all other cases, the MES operations personnel will arrange for evacuation.

Within the MES, the Health Coordination Centre manages cross-border emergencies and coordinates incidents that occur within Spanish territory. To enhance the management of cross-border disasters, and under the auspices of the Egalurg Project, the intervention protocols, jointly developed by Spain, France, and Andorra, were created. Alongside these, the GA-IMV platform facilitates victim registration, real-time translation, and information sharing between emergency teams and reference hospitals on both sides of the border. However, for these systems to be effective, adequate training is essential for personnel in both the MEUs and PEUs.

### 1.5. The present study

Current scientific literature includes several studies focused on the training and evaluation of PFA interventions. These studies address aspects such as the effectiveness of PFA, participant satisfaction, and changes in knowledge, attitudes, and practices following training or intervention [17]. However, no studies have been found that specifically address the coordination and management of PFA within prehospital emergency medical systems in crisis contexts.

In light of this research gap, the aim of the present study is to analyze a PFA training simulation integrated into a broader medical emergency simulation exercise following APCAT protocols. This exercise involves emergency healthcare personnel from three different countries, thus allowing for the examination of the implementation and management of PFA in the context of international collaboration. In this way, the study provides valuable insights into both the performance of PFA protocols and their coordination during emergencies within a cross-border, multi-agency, and multicultural environment.

## 2. Materials and methods

### 2.1. Participants

Seventy (70) healthcare responders participated in the simulation. Spain deployed five MEUs and one PEU, while France and Andorra contributed two and one MEUs, respectively. The PEU was composed of four psychologists who performed the training and evaluation tasks (1), coordination duties (1), and triage, registration, and psychological assistance (2).

Thirty-two (32) volunteers acted as the victims: eight (8) portrayed critical victims (RED zone), fourteen (14) represented victims with delayed emergency needs (YELLOW zone), eight (8) represented victims with minor injuries (GREEN zone), and two (2) represented the deceased (BLACK zone). Among the fourteen (14) victims located in the GREEN zone, thirteen (13) portrayed individuals with moderate levels of stress and anxiety, and one (1) represented a victim with a history of cardiovascular disease who suffered an acute anxiety crisis and required urgent evacuation. The average age of those in the GREEN zone was 21.07 years (*SD* = 7.79), with 71.43% being female.

### 2.2. Procedure and ethics

The simulation exercise took place in April 2022. It was based on a foreseeable risk scenario that involved a human stampede during a concert in the town of Llivia (Spain), which is located near the border with France and close to Andorra. The risk scenario was designed by the project's multidisciplinary team, which was composed of professionals from emergency medical services, psychology, civil protection, and public health from Spain, France, and Andorra. It was based on a foreseeable risk identified in prior safety assessments of large public events. Its design adhered to the recommendations of the Emergency Response Framework by the WHO, which guides international planning and response for health emergencies [31], the framework of the European Civil Protection and Humanitarian Aid Operations (ECHO), which provides strategies and guidelines for risk management and emergency responses within the European Union [32], the Territorial Emergency Plan of Catalonia (INFORCAT), which establishes the operational framework for comprehensive emergency management in the Spanish region of Catalonia [33], and the French national ORSEC plan (*Organisation de la Réponse de Sécurité Civile*), which organizes civil security responses to disasters and major emergencies in France [34].

The event attracted a young audience from all three regions. The scenario began when a singer suffered severe trauma after falling from the stage, thus leading to the sudden end of the concert. Chaos ensued during the evacuation, thus resulting in a human stampede. The disaster alert was issued by the medical unit (comprised of an ambulance and two emergency technicians), which was legally required to be present at any cultural or sporting event. This triggered the activation of the cross-border disaster protocols. In accordance with the protocols developed under the Egalurg project, the Spanish MES was responsible for managing the health emergency, in collaboration with Emergency Units from France and Andorra. No volunteers, including those from the Red Cross or other organizations, took part in the simulation.

Following the arrival of the first MEU, the health area was established, and the START triage commenced. Subsequently, the PEU set up its base in the GREEN zone. A nurse from one of the MEUs contributed by France was assigned as support staff to the PEU. The PFA protocol was applied. As established by the protocols, the PEU lead acted as a liaison with the MEUs deployed to the area, thus monitoring and intervening when necessary to protect the mental health of the healthcare personnel. Advanced selection and evacuation orders to appropriate health centers were executed through the GA-IMV Platform for those physically affected. As the PEU lacked network access, triage and registration were conducted using cards.

The healthcare workers were members of the aforementioned MES and were activated by their respective command centers. The simulation formed part of their training program. The participants who acted as victims were volunteers. The inclusion criteria required the participants to be over 18 years old and free from any disabling conditions. There were no further exclusion criteria. They received no compensation for their participation. Prior to taking part, they were briefed on the study's objectives, data confidentiality, anonymity, and their right to withdraw at any time. The participants provided informed consent, thus agreeing to the terms of the study.

Subsequently, both the healthcare personnel and the volunteers received role-specific training before the scheduled simulation exercise. In particular, the training of the PEU coordinator and those responsible for triage, registration, and evaluation was conducted by the psychologist in charge of training and evaluation within the PEU. These psychologists are part of the staff of the regional emergency medical services and had more than one year of experience in delivering PFA in emergency situations. Therefore, on the day prior to the simulation exercise, they received a two-hour training session exclusively focused on the APCAT protocols that were to be implemented and tested during the exercise. Additionally, the psychologist responsible for training and evaluation trained the volunteers acting as victims. The other psychologists in the PEU did not participate in this training process and were unaware of the preparation provided to the actors. The training lasted two hours and took place on the same day the simulation was conducted. None of the volunteers had previously performed the assigned roles. These volunteers were coached to display typical anxiety symptoms associated with a normal stress response, such as nervousness, difficulty concentrating, restlessness, rapid or shallow breathing, and expressions of worry or fear. This approach aimed to ensure realistic and consistent portrayals of psychological distress. In total, seven volunteers were trained to exhibit mild, normal stress reactions to a disaster situation, while six were instructed to display moderate levels of psychological symptoms. Only one volunteer was specifically trained to simulate an acute anxiety episode, combined with a pre-existing cardiac condition, to add a layer of complexity to the scenario. The acute stress reaction was portrayed through a combination of physical and emotional symptoms that were easy to represent, such as difficulty breathing, palpitations, disorientation, and intense fear, accompanied by agitated behavior and verbal expressions of confusion and heightened anxiety, thus providing a credible and realistic recreation of the condition. The simulation was conducted on the designated day and time. All protocols adhered to the Declaration of Helsinki and were approved by the Institutional Review Board of the Medical Emergency Services of Catalonia (Spain).

### 2.3. Information collected and data analysis

Demographic data of the simulated casualties were collected by the PEU members using a standard Affiliation Card. The competences evaluated were as follows: psychological emergency management, teamwork, problem-solving ability, multicultural context management, roles and functions, and follow-up of ethical principles. During the simulation, the person responsible for the PEU training monitored and evaluated the competencies of the members of their PEU. At the end of the drill, both the PEU psychologists and those who acted as victims completed a form that requested a subjective and qualitative evaluation of these competences. To this end, four closed-ended questions were included to assess the psychological emergency management, teamwork, problem-solving ability, and management of the multicultural context on a scale from 0 to 5 (i.e., very poor, poor, fair, good, or very good). Two dichotomous closed-ended questions were used to evaluate the clarity of roles and functions of the participants and the adherence to the ethical and deontological principles of the profession. Three open-ended questions invited the respondents to briefly describe their overall impression of the exercise, what they liked and disliked, and a final section allowed them to suggest improvements. Quantitative data were subjected to descriptive and prevalence statistical analyses using the SPSS software, version 29.0. Qualitative data were reviewed and analyzed to identify the strengths and weaknesses and to propose improvements. For the qualitative analysis of the questionnaires, a thematic framework was used based on the previously referenced competencies, which had been selected prior to the study by consensus among the main project leads: the PEU psychologist responsible for training and evaluation tasks (OM), the coordinator of the Egalug project (YL), and the head of psychological services of the emergency medical system of the Catalonia region in Spain (AC). This framework served as a guide to categorize the comments. No qualitative analysis software was used. The collection of comments by competency was conducted by the PEU psychologist (OM). A thematic content analysis of these comments and the grouping of similar responses under a common representative item were performed by consensus among the three aforementioned leads. In the results table, one or more items are presented for each competency, depending on the quantity and richness of the data collected, thereby reflecting the diversity of observations from the evaluator, psychologists, and victims.

## 3. Results

The PFA protocol was implemented and the psychologist performance, coordination and management of PFA were evaluated. Strengths and weaknesses were identified, thus enabling the formulation of improvement suggestions. The results are summarized in Table 1.

**Table 1.** Assessment of PEU Competencies during the Simulation: Strengths, Weaknesses, and Improvement Proposals.

**Competence**	**Assessment**	**Strengths**	**Weaknesses**	**Improvement Proposals**
Psychological Emergency Management	1: Correct to good. 2: Average 4.36 (*SD*: 0.72) out of 5. 3: No deviations in victim care. Failures in triage and affiliation.	1: Applied training. 2: Received psychological attention. 3: Immediate response to victims.	1: Communication problems. 2: Lack of basic resources. 3: Issues in triage and affiliation.	1: Improve communication and coordination with other teams. 2: Provide basic resources (as water, shaded area, etc). Improve response speed. 3: Improve triage and affiliation.
Teamwork	1: Correct to good. 2: Average 4.21 (*SD*: 0.67) out of 5. 3: Lack of prior assessment.	1: Good communication among interveners. 2: Good communication among professionals. 3: Communication among interveners.	1: Lack of prior assessment of the situation. 2: Lack of information about the situation. 3: Absence of prior assessment of the situation.	1: Provide basic resources. Improve response speed. 2: Improve coordination among interveners. 3: Enhance prior situation assessment, strengthen communication among teams.
Problem-Solving Ability	1: Correct to good. 2: Average 4.14 (*SD*: 0.74) out of 5. 3: No deviations in solving psychopathological issues. Problems in addressing basic needs.	1: Good ability to solve psychopathological problems. 2: No deviations in psychopathological resolution. 3: No deviations in resolution capability.	1: Delay or lack of attention to basic needs. 3: Problems in addressing basic needs.	1: Expedite the provision of basic needs. 3: Strengthen attention to basic needs.
Multicultural Context Management	1: Correct to good. 2: Average 4.21 (*SD*: 0.86) out of 5. 3: No deviations in multicultural psychological care. Lack of adequate data record.	1: Adequate multicultural psychological care. 2: Adequate multicultural psychological care. 3: Multicultural care without deviations.	1: Absence of adequate data record. 3: Lack of information on cultural aspects.	1: Implement adequate data record for multicultural issues. Provide training on cultural aspects. 3: Strengthen multicultural care. Ensure adequate multicultural data registration.
Roles and functions	1: Consider their role clear. 2: 7.14% consider their role do not clear. 3: Identified task interchange without awareness.	1: Clarity of roles. 2: Some don't consider their roles clear. 3: Unintentional task interchange.	1: No identification of task interchange. 2: Role confusion. 3: Unintentional task interchange	1,2,3: Reinforce role clarity.
Follow-up of Ethical Principles	1,2: Consider ethical principles followed. 3: No deviations in this point.	1,2: Adherence to ethical principles. 3: No deviations identified.	1,2,3: No deviations identified.	1,2: Maintain adherence to ethical principles. 3: Ensure compliance with regulations.

Note: 1: PEU psychologist; 2: Simulated casualties; 3: Supervisor.

Unfortunately, the need for PFA at a safe area nearby the disaster site for the emergency healthcare personnel was not anticipated during the planning phase, which prevented data collection from this segment of the population.

## 4. Discussion

As indicated, the PFA protocol was implemented, and both the management and coordination of the PEU within the cross-border mass casualty incident simulation were analyzed. Strengths and weaknesses were identified, and suggestions for improvement were proposed. However, a planning oversight prevented the collection of data from the healthcare personnel (none was acting as victims). As a result, the simulation exercise only partially achieved its stated objectives.

No studies similar to the present one has been identified, thus precluding a discussion in relation to previous findings The analysis and discussion of results by area is described below.

### 4.1. Psychological emergency management

The data indicate that the psychological management of the emergency was generally assessed as correct to good. This positive perception suggests that the training received by the PEU personnel was effective and appropriately applied in the simulated context. The immediate response and the ability to provide psychological support were regarded as key strengths. However, deficiencies were identified in structural aspects, particularly in inter-team coordination and in the implementation of triage and registration. A more systemic approach is necessary, one that connects the work of the PEU with the digital platform for registration and triage. Additionally, the lack of basic resources (such as water, blankets, shade, etc.) suggests insufficient planning of the intervention setting, which may compromise the well-being of both the victims and the responders. This discrepancy between the quality of care and operational processes highlights a mismatch between the team's clinical competencies and the logistical functions of the system. All evidence suggests that effective psychological support is not sufficient unless it is properly integrated with the rest of the operational framework.

### 4.2. Teamwork

The results indicate a generally positive evaluation of teamwork. One of the main identified strengths was the quality of communication between the PEU psychologists and the victims. This reflects a solid foundation of mutual understanding and cooperation, which are essential in high-pressure emergency contexts. However, an unclear initial guidance of the PEU personnel was recorded. Such deficiency may have contributed to moments of miscoordination and reduced the capacity of teams to act swiftly and efficiently during the simulation. The findings point to a need for targeted improvements in preparatory procedures. Specifically, enhancing the assessment of the situation prior to deployment is critical. This includes not only gathering and disseminating contextual information but also clarifying roles and responsibilities among all involved teams. Furthermore, strengthening the coordination mechanisms among interveners is a vital step toward improving the overall effectiveness of teamwork in future exercises. In conclusion, while internal communication and professional collaboration are clear strengths, their full potential can only be realized through more structured and comprehensive pre-incident planning.

### 4.3. Problem-solving capacity

The data indicate a strong capacity among PEU personnel to address mental health issues. This reflects a good level of professional training and an effective application of the PFA protocol. However, shortcomings were identified in meeting the victims' basic needs (such as food, hygiene, and shelter). This imbalance suggests an excessive focus on the clinical dimension of the intervention, to the detriment of a more holistic view of the affected individuals' well-being. Basic needs are essential for the effectiveness of any initial psychological intervention, as they influence the perception of safety and emotional containment. Therefore, it may be appropriate to strengthen the PFA protocol by integrating basic humanitarian standards into its operational deployment, thus ensuring that the psychological team acts not only as a clinical agent but also as a guarantor of a safe and supportive environment.

### 4.4. Managing the multicultural context

Psychological support in multicultural contexts was positively evaluated, with no significant deviations reported for the direct interactions with victims. This strength suggests an adequate level of cultural sensitivity among the team members, despite the lack of explicit training in this area. However, the absence of a systematic record of cultural data was noted, thus limiting the ability to document and tailor interventions to diverse profiles. Additionally, a language barrier in the green zone—where the emergency technician only spoke French—hindered coordination and mutual understanding. In this regard, effective multicultural care cannot solely rely on the individual competence of professionals; it must be institutionalized through specific training, appropriate data collection systems, and, in cross-border contexts, the availability of translators or intercultural mediators.

### 4.5. Clarity of roles and responsibilities

Although roles had been previously assigned, operational confusion was observed at the beginning of the simulation within the PEU team. The PEU was composed of four psychologists with differentiated responsibilities: training and evaluation (1), coordination (1), and triage, registration, and psychological support (2). Although individual roles were previously assigned and understood, during the initial phase of the simulation, the psychologist responsible for coordination and the two in charge of triage, registration, and psychological support temporarily engaged in overlapping tasks, without clearly adhering to their specific assignments. They only assumed their designated roles after being prompted by the training and evaluation lead. Moreover, redundancies in triage activities were observed and reported by several actors (victims). Furthermore, one of the victims required evacuation during the exercise. Although the PEU's internal communication protocols were clearly defined, the liaison officer assigned by the emergency coordination team had not been properly briefed on them, which led to coordination issues and required further intervention by the training and evaluation lead. This lack of clarity may stem from insufficient planning or the absence of a common operational framework that integrates individual functions within the team and with other emergency units. Role ambiguity can lead to duplication, omissions, or interprofessional tensions, especially in high-pressure situations such as a mass casualty incident. While prior training was positively regarded, the results suggest it should be complemented with practical exercises that simulate real challenges in interagency coordination. A clarity of functions should be reinforced through visual protocols, role maps, and supervised simulations with role rotation, thus promoting the practical internalization of each professional's responsibilities and limits.

### 4.6. Adherence to ethical principles

This dimension was the most consistently positive. The responders, victims, and the observer agreed that ethical principles were respected and no deviations were reported. This reinforces the impression that PEU professionals have fully internalized the basic principles of their code of ethics and apply them competently, even in simulated contexts. Although no immediate improvements were identified in this area, it is advisable to maintain training spaces that encourage ethical reflection in crisis situations, particularly when facing common dilemmas in the simultaneous care of multiple victims.

### 4.7. Strengths and limitations of this study

This study presents several strengths. Notably, it is the first to document a full-scale simulation that involved PEUs within a cross-border disaster context, thus providing valuable empirical insights in an area with limited literature. Given the nature of the study that involved a single PEU and a small, non-random sample, the statistical generalizability of the findings is limited. However, the results offer valuable insights that may be transferable to similar cross-border disaster response contexts. Transferability refers to the extent to which findings can be applicable or relevant to other settings with comparable conditions, thus allowing readers and practitioners to judge the usefulness of these results within their own contexts. The demographic composition, which was skewed towards young females, may not accurately reflect real-world victim populations.

Furthermore, the use of self-reported measures may introduce social desirability or recall biases, that is, this study heavily relied on self-reported evaluations by the PEU psychologists regarding their own competencies, as well as feedback from the volunteers who acted as victims concerning the care received. Such subjective assessments are subject to biases that may affect the objectivity of the results. On the one hand, social desirability bias may lead the participants to provide more favorable or socially acceptable responses, thus influencing the accuracy of the information given. On the other hand, the Hawthorne effect implies that the participants may alter their behavior simply because they know they are being observed, which could affect the natural dynamics during the study. These biases should be carefully considered. Future research should contemplate the use of more objective measures or external evaluations to complement self-reports and strengthen the validity of the findings.

However, the most significant limitation was that the simulation planning did not consider the possibility that healthcare and rescue personnel might require psychological support. As a result, the PEU did not activate the PFA protocol for this group, thus preventing the testing of this key component. This omission is particularly relevant given that one of the core rationales of PFA is to support the supporters. As such, the absence of implementation and the evaluation of PFA for responders not only restricts the scope of the exercise but also limits the comprehensiveness of the study's conclusions regarding the overall effectiveness of the protocol in this simulated context. Future simulations should explicitly address this aspect to enable a more complete and representative assessment of PFA in operational settings. Although this does not affect the validity of the obtained results, it does restrict the scope of the exercise, as the intervention was not evaluated with one of the priority groups defined in the PFA protocol.

Another notable limitation was the failure of the PEU to access the GA-IMV digital platform due to a lack of network connectivity. This technical issue prevented the team from using the intended digital tools for registration, triage, and coordination, thus restricting the assessment of the integrated digital response system. As a result, the simulation inadvertently shifted to a fallback scenario that relied on analogue methods, thus limiting the evaluation of the platform's interoperability and its role in facilitating coordinated care across emergency units. This incident underscores the importance of ensuring reliable digital infrastructure and contingency planning in future simulation exercises that involve e-health tools.

### 4.8. Implications and future directions

Based on the results and identified limitations, future studies should consider including a larger number of participants and ensuring a more diverse sample to improve the generalizability of findings to real-world cross-border interventions in mass casualty incidents. Moreover, it would be pertinent to replicate the study with different PEUs to assess consistency in the implementation of the PFA protocol and to explore potential variations in its effectiveness across teams or regions. Another priority is to design simulations that explicitly include the activation of the PFA protocol for healthcare and rescue personnel, thus allowing for the evaluation of this key component from the perspective of the responders themselves. Additionally, it is recommended to complement self-report measures with objective tools, such as external observations, behavioral indicators, or third-party assessments, to mitigate biases such as social desirability and inaccurate recall. Finally, it is recommended that the PEU have access to the GA-IMV digital platform in future simulations in order to fully assess the integration, coordination, and utility of digital tools in real-time disaster response scenarios.

## 5. Conclusions

Overall, this study provides valuable empirical evidence in a still underexplored area and highlights the usefulness of simulations as a fundamental tool to evaluate and refine psychological intervention protocols in disaster contexts. The simulation demonstrated the performance and applicability of the tested protocols, but also revealed significant operational, logistical, and coordination weaknesses. These findings emphasize the need for a more systemic integration of the PFA protocol within emergency systems, thus strengthening both humanitarian and organizational aspects, especially in cross-border contexts. The lessons learned reinforce the need to design more realistic and inclusive exercises that allow for the evaluation of all components of the PFA, including support for the responders themselves. These insights offer clear guidance for future research and the development of more effective protocols for real cross-border emergency scenarios.

## Use of AI tools declaration

The authors declare they have not used Artificial Intelligence (AI) tools in the creation of this article.
